# OA21 An Interesting Case of Late Onset PFAPA Overlapping with Bechet’s Disease

**DOI:** 10.1093/rap/rkae117.021

**Published:** 2024-11-05

**Authors:** Kapil Garg, Anupama Nandagudi

**Affiliations:** Basildon University Hospital, Mid and South Essex NHS Foundation Trust, Basildon, United Kingdom; Basildon University Hospital, Mid and South Essex NHS Foundation Trust, Basildon, United Kingdom

## Abstract

**Introduction:**

Periodic fever, aphthous stomatitis, pharyngitis, and adenitis (PFAPA) syndrome is most common periodic fever syndrome in childhood. The median age of onset is 11 months and attacks cease by age 10. It’s a diagnosis of exclusion made on clinical grounds after excluding recurrent infections, lymphoma, IBD, cyclic-neutropenia and auto-immune conditions. Diagnostic criteria include regular recurring febrile episodes and presence of at least one of 3 features (aphthous stomatitis, pharyngitis, adenitis). PFAPA is benign condition with no long-term sequelae but may cause significant morbidity during flares. We present a case of PFAPA with Bechet’s disease Overlap and delayed onset beyond childhood.

**Case description:**

We present case of 29-year-old Caucasian female with Spanish maternal ancestry. She underwent left hip replacement for Perthes disease previously. She was admitted at age of 17 years with acute onset high grade fever, tonsilitis, macular erythematous rash, polyarthralgia, high inflammatory markers with raised WCC, CRP of 100 and ferritin of 785. She was treated with empirical antibiotics with no response and had negative cultures. There were no features of CTD with negative ANA, RF, CCP and complements. She was followed-up in rheumatology clinic with possibility of stills disease. She continued to get flares of fever, painful mouth ulcers, and joint pains. She would get 6 weekly episodic flares each lasting 5-10 days and would develop mouth ulcer >1.5cm on lower lip with necrotic base, anterior cervical and submandibular lymphadenopathy accompanies with very high inflammatory markers. There were no other systemic, vascular or ocular features although she did suffer with migraines. Due to poor response with oral steroids, methotrexate was initiated considering possibility of inflammatory/reactive arthritis. She started to develop painful genital ulcers inside labia majora with her usual flares by age 18. At age 20 she was reviewed at tertiary centre and underwent genetic testing for hereditary auto-inflammatory syndromes, which was negative. She was diagnosed as Bechet’s disease and PFAPA syndrome (Periodic fever with aphthous stomatitis, pharyngitis, and adenitis) overlap. Her HLA-B51 was negative. Methotrexate was stopped and colchicine 1 mg daily was continued. Azathioprine was introduced and optimised to 150 mg daily. Patient is on regular follow-up in rheumatology and continues to get flare ups although they have become milder in intensity and less frequent occurring 2 monthly. She has not developed any long-term complications, major organ involvement or vascular thrombosis so far.

**Discussion:**

The flares in our patient were treated over the years as infections, post-streptococcal arthritis and still’s disease. There was periodicity in the episodes of fever, mouth ulcers, sore-throat, cervical adenitis and joint pains, happening every 6 weeks for initial few years. There would be completely asymptomatic intervals in between flares with normalisation of inflammatory markers. The key is ‘periodicity’ of symptoms in periodic fever syndromes, which was not recognised during early years in our patient. Patient was rightly referred for genetic testing which ruled out monogenic auto-inflammatory conditions.

PFAPA syndrome has overlapping clinical features with Bechet’s Disease and may reflect an early phenotype of BD. PFAPA usually resolves by age 10 or post-tonsillectomy in majority of children and would rarely present in adulthood. This could explain delayed recognition of this syndrome by adult rheumatologists due to less experience with similar conditions. Our patient had partial response to oral steroids and azathioprine and colchicine were introduced as prophylaxis therapies. PFAPA had no long-term sequalae although same cannot be commented about Bechet’s disease. Our patient has not developed any major organ or ocular involvement so far. As our patient continues to get episodes of PFAPA, we might consider IL-1 inhibitors such as anakinra or canakinumab going forwards. Canakinumab was not approved in her case previously.

**Key learning points:**

• There are genetic similarities between recurrent aphthous ulcers, Bechet’s disease and PFAPA syndrome and these conditions could be grouped under ‘Bechet’s Disease Spectrum (BDS)’ with recurrent aphthous ulcers being mildest, PFAPA syndrome moderate and Bechet’s disease as the most severe phenotype.

• Early diagnosis addressed patient and family anxiety, facilitates early initiation of treatment avoiding unnecessary investigations, antibiotics and hospitalisation during flares. Family history is paramount while assessing periodic fever patient as many of these have associated familial predispositions. There are no diagnostic tests for PFAPA syndrome.

• HLA-B51 is risk allele for BDS but is not diagnostic by itself. Hence the diagnosis of PFAPA syndrome hinges on exclusion of commoner conditions.

• Prednisolone 1-2 mg/kg given as soon as possible at onset of flare relieves the symptoms of fever, arthralgia and malaise. Majority of patient do not require second dose of prednisolone. Mouth ulcers take longer to recover. Colchicine is used as preventive medicine to reduce the frequency of flares. Interestingly tonsillectomy may lead to complete resolution of PFAPA symptoms in children. Interleukin (IL-1) antagonists such as anakinra and canakinumab have proven to be beneficial in small studies. Evidence also suggests maintaining vitamin D levels around 100nmol/L helps to decrease in the number and duration of flares in PFAPA.

• More controlled and larger studies are required for further evidence.

• Recognition of PFAPA as a continuum of Behcet’s spectrum is important as they share certain common clinical features. PFAPA may reflect an early phenotype of Bechet’s disease occurring in early childhood.

